# A phylogeny of the evening primrose family (Onagraceae) using a target enrichment approach with 303 nuclear loci

**DOI:** 10.1186/s12862-023-02151-9

**Published:** 2023-11-17

**Authors:** Rick P. Overson, Matthew G. Johnson, Lindsey L. Bechen, Sylvia P. Kinosian, Norman A. Douglas, Jeremie B. Fant, Peter C. Hoch, Rachel A. Levin, Michael J. Moore, Robert A. Raguso, Warren L. Wagner, Krissa A. Skogen, Norman J. Wickett

**Affiliations:** 1 Arizona State University, PO Box 875502, Tempe, AZ 85287 USA; 2 Texas Tech University, Box 43131, Lubbock, TX 79409 USA; 3 Negaunee Institute for Plant Conservation Science and Action, Chicago Botanic Garden, 1000 Lake Cook Rd, Glencoe, IL 60022 USA; 4 Whitehead Institute for Biomedical Research, 455 Main St, Cambridge, MA 02142 USA; 5 Department of Ecology and Evolutionary Biology, University of Arizona, PO Box 210088, Tucson, AZ 85721 USA; 6 University of Florida, 220 Bartram Hall, Gainesville, FL 32611 USA; 7 Northwestern University, 2205 Tech Dr, Evanston, IL 60208 USA; 8 Missouri Botanical Garden, 4344 Shaw Blvd, St. Louis, MO 63110 USA; 9 Amherst College, 25 East Dr, Amherst, MA 01002 USA; 10 Oberlin College, 119 Woodland St, Oberlin, OH 44074 USA; 11 Cornell University, 215 Tower Rd, Ithaca, NY 14853 USA; 12 Smithsonian Institution, MRC-166, PO Box 37012, Washington, DC 20013 USA; 13 Clemson University, 132 Long Hall, Clemson, SC 29634 USA

**Keywords:** Phylogenomics, HybSeq, Onagraceae, Evening primrose, Target enrichment, Plant systematics, Phylogenetics

## Abstract

**Background:**

The evening primrose family (Onagraceae) includes 664 species (803 taxa) with a center of diversity in the Americas, especially western North America. Ongoing research in Onagraceae includes exploring striking variation in floral morphology, scent composition, and breeding system, as well as the role of these traits in driving diversity among plants and their interacting pollinators and herbivores. However, these efforts are limited by the lack of a comprehensive, well-resolved phylogeny. Previous phylogenetic studies based on a few loci strongly support the monophyly of the family and the sister relationship of the two largest tribes but fail to resolve several key relationships.

**Results:**

We used a target enrichment approach to reconstruct the phylogeny of Onagraceae using 303 highly conserved, low-copy nuclear loci. We present a phylogeny for Onagraceae with 169 individuals representing 152 taxa sampled across the family, including extensive sampling within the largest tribe, Onagreae. Deep splits within the family are strongly supported, whereas relationships among closely related genera and species are characterized by extensive conflict among individual gene trees.

**Conclusions:**

This phylogenetic resource will augment current research projects focused throughout the family in genomics, ecology, coevolutionary dynamics, biogeography, and the evolution of characters driving diversification in the family.

**Supplementary Information:**

The online version contains supplementary material available at 10.1186/s12862-023-02151-9.

## Background

**Fig. 1:**
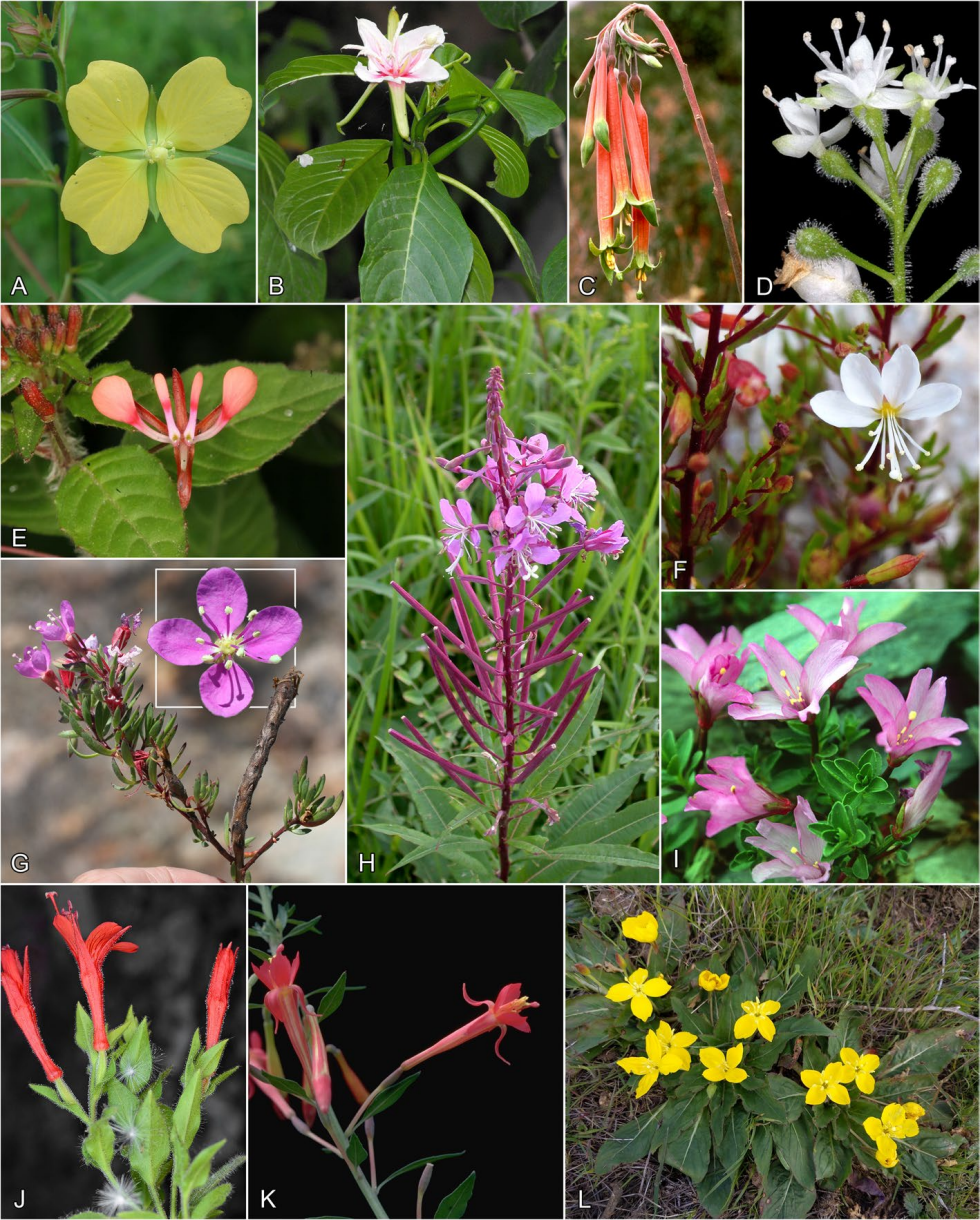
Onagraceae. subfamily Ludwigioideae. **A** *Ludwigia octovalvis*. Flower and immature capsule. Baldwin Co., Alabama (image Warren Wagner in 2003). Subfamily Onagroideae B-L. **B** Tribe Hauyeae. *Hauya elegans* subsp. *lucida*. Branch with flower at anthesis and in bud. Cultivated at San Diego Zoo (image Wikipedia in 2008). **C**, **D** Tribe Circaeeae. C. *Fuchsia inflata*. Inflorescence. Cusco, Peru (image P. Berry in 1978). **D** *Circaea pacifica*. Inflorescence and young fruit. Benton Co Oregon (image G. Carr-4162b in 2006). **E**, **F** Tribe Lopezieae. **E** *Lopezia racemosa* subsp *racemosa*. Flower with inflorescence and leaves. Puebla, Mexcio (image Jon Amith in 2007). **F** *Megacorax gracielanus.* Chasmogamous flower with bud and stem with leaves. Sierra de Coneto, Durango, Mexico (image M. Socorro Gonzalez-Elizondo in 2021). **G** Tribe Gongylocarpeae. *Gongylocarpus fruticulosus*. Stem with flowers and gall-like mature fruit embedded in pith of stem, indehiscent, and inset showing flower closeup. Isla Magdalena, Baja California Sur, Mexico (images Jon Rebman in 2016). Tribe Epilobieae **H**-**J** H. *Chamaenerion angustifolium* subsp. *circumvagum*. Stem with inflorescence with open flowers and maturing capsules. MN (image Peter M. Dziuk, Minnesota Wildflowers in 2002). **I** *Epilobium nankotaizanense*. Habit with flower. Taiwan (image Ching-I Peng in 2008). **J** *Epilobium canum* subsp *latifolium*. Stem with flowers and some dispersed seeds. Curry Co., OR (image Gerry Carr 6317b in 2015). Tribe Onagreae. **K**, **L** K. *Xylonagra arborea*. Stem with flowers and very young fruit. Cultivated at Missouri Botanical Garden, originally from Baja California, Mexico (image Ching-I Peng in 1982). L. *Taraxia ovata*. Habit with flowers. Monterey, CA (image Christian Schwarz)

**Fig. 2:**
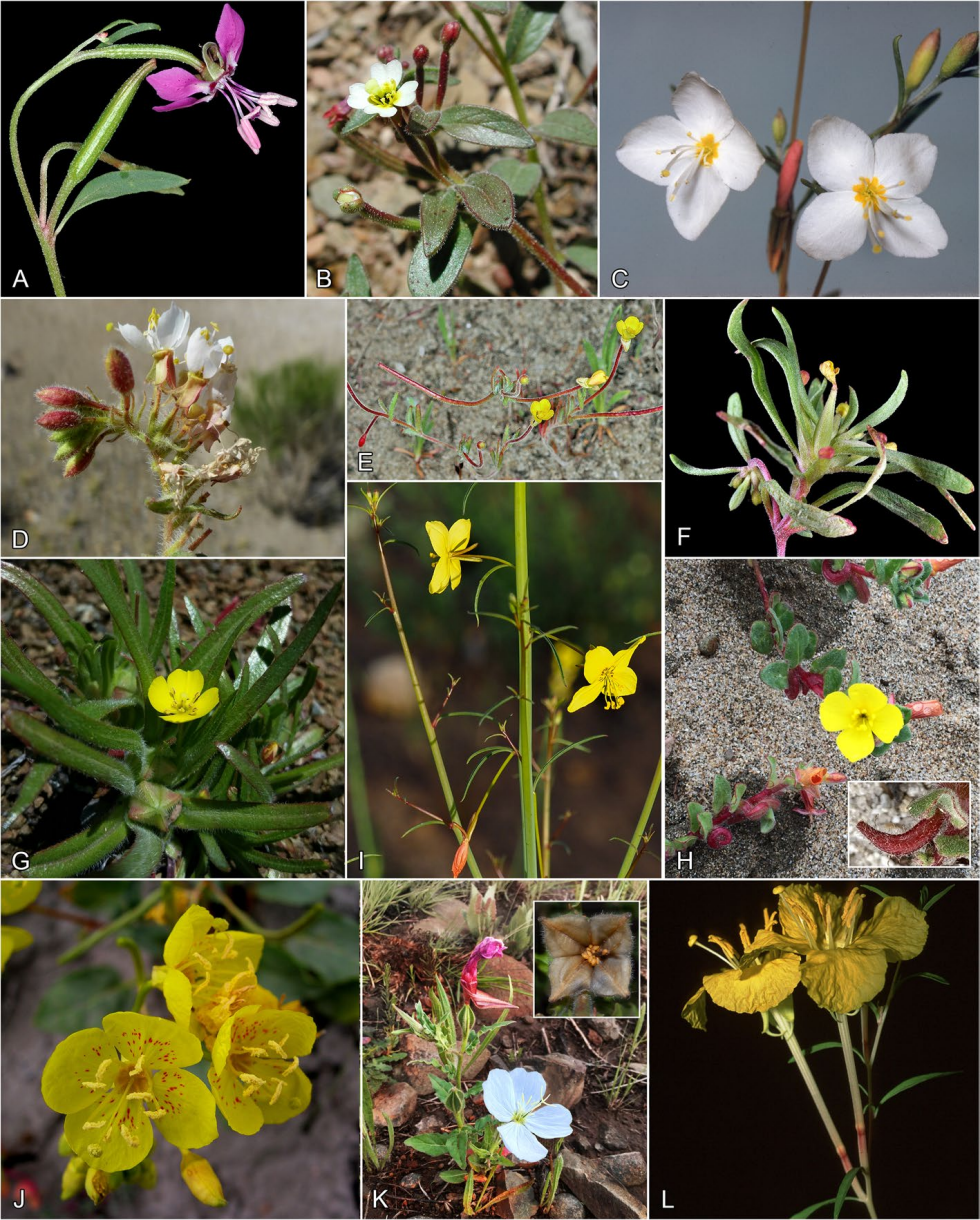
Tribe Onagreae. A-L. **A** *Clarkia rhomboidea*. Stem with flower and immature capsule. Jefferson Co., OR (image Gerry Carr 6468b in 2006). **B** *Chylismiella pterosperma*. Stem with leaves, flower, and buds. Inyo Co., CA (image Larry Blakely in 2001). **C** *Gayophytum diffusum* subsp. *diffusum*. Stem with flowers and buds. Tulare CA (image Peter Raven collection). **D** *Eremothera boothii* susbp. *boothii*. Flowers and buds. Mono Co., CA (image Michael Moore 3260 in 2015). **E** *Camissonia contorta*. Plant with flowers and young capsules. Klickitat Co., WA (image Gerry Carr 0356b in 2009). **F** *Neoholmgrenia andina*. Plant with fading flower and immature capsule. Harney Co., OR (image Gerry Carr 0996b in 2011). **G** *Tetrapteron graciliflorum*. Plant with flower and immature capsule. Marin Co., CA (image David Greenberger in 2018). **H** *Camissoniopsis cheiranthifolia* subsp. *cheiranthifolia*. Stem with flowers and immature capsules. San Mateo Co., CA (image Leslie Flint in 2015). Inset of immature capsule. Monterey, CA (image Steve Rovell in 2016). **I** *Eulobus californicus*. Stem with flowers. San Diego Co., CA (image Ron King in 2021). **J** *Chylismia eastwoodiae*. Flowers and buds with capsules in background. Emery Co., UT (image Rob Raguso in 2001). **K** *Oenothera tetraptera*. Plant with flower (from Oageng Modise) and immature capsule. South Africa (image Behrens in 2021). Inset of dehisced capsule with seeds clustered inside. South Africa (image Warren Wagner in 2008). **L** *Oenothera toumeyi*. Stem with flowers. Cochise Co., AZ (image Rob Raguso in 1996)

The evening primrose family (Onagraceae, Myrtales) comprise 664 species of herbs, shrubs, and trees across 22 genera [1], with almost two-thirds of the species occurring in tribes Epilobieae (2 genera, 173 spp.; Fig. 1H-J) and Onagreae (13 genera, 265 spp.; Figs. 1L–K and 2A–L). Onagraceae have a cosmopolitan distribution, with the majority of species concentrated in the Americas, especially western North America. Almost all genera in the tribes Lopezieae, Gongylocarpeae, Epilobieae, and Onagreae are endemic to or have had their major basal radiation in the Madrean Floristic Region of southwestern North America [2]. Members of *Epilobium* and *Chamaenerion* (the nomenclaturally correct name for what has previously been referred to by the synonym *Chamerion*) (Fig. 1H–J), for example, have wind-borne seeds and are distributed widely across the world [3, 4]. *Fuchsia* (Fig. 1C)*,* with animal-dispersed berries, most likely arose in South America or southern North America and diversified extensively in the Andean region but has also colonized New Zealand and Australia (no longer extant), as well as isolated Tahiti [4–8]. Since the mid-twentieth century, the family has been developed as a model system for studying plant evolution [9]. However, a limitation of these previous studies has been the absence of a robust phylogenetic framework within which to examine the evolution of these traits.

Within Onagraceae, there is a wide range of ecological specialization, pollination syndromes, breeding systems, and chromosomal organization, as well as striking inter- and intraspecific variation for floral scent [4]. The family includes lineages with hummingbird pollination as well as lineages of presumably ancestral vespertine anthesis and hawkmoth pollination with multiple evolutionary origins of bee pollination and especially autogamy [4]. Permanent translocation heterozygosity (PTH), which results in the severe attenuation of recombination during meiosis and is extremely rare in plants, occurs in a single species of *Gayophytum*, is quite common in *Oenothera* (46 spp.), and is thought to be a major modulator of the evolutionary and ecological dynamics within *Oenothera* [10, 11]. In addition, polyploidy is common throughout the family, with an estimated 39% of species being polyploid [4]. Despite its modest size, the family has played a major role in evolutionary theory, starting with De Vries’ rediscovery of Mendel’s laws through experimentation with *Oenothera*, leading to ideas crucial to the development of the Modern Synthesis [12]. More recent research in the group has focused on themes ranging from cytology, embryology, palynology, chemistry, and reproductive and pollination biology [1, 9], chromosome evolution [10, 13–15], and the role that trade-offs in reproductive mode, floral morphology, and floral scent play in driving diversification in the context of plant-insect interactions [16–19].

Onagraceae systematics has a long history of detailed comparative work, with the most recent family-wide treatment [1] synthesizing all available morphological and phylogenetic evidence. The family consists of two subfamilies [1]: Ludwigioideae, comprising *Ludwigia* (82 spp.; Fig. 1A), and Onagroideae, with all remaining taxa. Onagroideae is currently subdivided into six tribes [1]: Hauyeae (1 genus, 2 spp.; Fig. 1B), Circaeeae (2 genera, 117 spp.; Fig. 1C, D), Lopezieae (2 genera, 23 spp.; Fig. 1E, F), Gongylocarpeae (1 genus, 2 spp.; Fig. 1G), Epilobieae (2 genera, 173 spp.; Fig. 1H–J), and Onagreae (13 genera, 265 spp.; Fig. 1K, L and  2A–L). Phylogenetic evidence based on targeted gene sequencing of plastid DNA [20] and plastid + nuclear DNA [21] confirmed the monophyly of the family and the individual monophyly of those tribes from which multiple species were sampled. Strong support was also found for *Gongylocarpus* (previously embedded within Onagreae) as sister to Onagreae + Epilobieae, spurring its subsequent elevation to the tribal level [1]. Within Onagreae, Levin et al. [21] demonstrated that *Oenothera* and *Camissonia* were not monophyletic as circumscribed at the time. Thus, Wagner et al. [1] subsequently expanded *Oenothera* to include the former genera *Calylophus*, *Gaura,* and *Stenosiphon*, and divided *Camissonia* into nine genera. Levin et al. [21] additionally found strong support for two deep lineages within *Oenothera*, referred to as lineages “A” and “B”, with the relationships among most genera in Onagreae and most sections within *Oenothera* poorly resolved.

Subsequently, Johnson et al. [17] inferred phylogenetic relationships of Onagraceae, with a focus on tribe Onagreae. They incorporated data from Levin et al. [21] while also expanding species sampling and adding two additional nuclear markers. In agreement with Levin et al. [21], Johnson et al. [17] found support for the monophyly of Onagreae, Epilobieae, and the recently erected Gongylocarpeae, as well as the previously detected lineages A and B within *Oenothera*. However, several conflicting hypotheses of relationships exist between the two analyses. For example, Levin et al. [21] found moderate support for the monotypic Baja California endemic *Xylonagra arborea* as sister to the rest of Onagreae. In contrast, the analysis of Johnson et al. [17] indicated weak support for this species being nested well within the tribe, with weak support for *Taraxia* as sister to the remaining members of Onagreae. These differences between the two studies suggest potential conflict among gene trees or other analytical constraints.

### Goals of the study

Several phylogenetic relationships within the subfamily Onagroideae remain unresolved. The individual monophyly of the subfamily’s six tribes as currently circumscribed appears strongly supported by morphology and DNA ( [1] and references therein), but relationships among them are not fully resolved. Additionally, most relationships within the species-rich Onagreae are equivocal, suggesting rapid diversification in this group. Here we employ targeted enrichment of 303 nuclear genes to: (1) elucidate relationships among tribes within Onagroideae, (2) understand relationships among genera in tribe Onagreae and within *Oenothera*, and (3) examine support for the monophyly of genera and historically difficult to resolve clades by exploring levels of conflict among gene trees. The phylogenetic resource provided here will be valuable for understanding biogeographic patterns in Onagraceae, as well as comparative studies ranging from trait evolution to comparative genomics to community ecology.

## Results and discussion

### Target capture and phylogenomic datasets

We used a target capture array of 322 low-copy nuclear protein-coding genes [22, 23] designed using transcriptomes of *O. serrulata* and *O. capillifolia* subsp. *capillifolia* (sect. *Calylophus*), from the 1KP Project [24]. The array uses 120-bp RNA probes to hybridize with genomic DNA fragments prior to amplification and sequencing. Attrition of target loci due to unknown causes in the laboratory, as well as subsequent bioinformatic quality filtering, resulted in a final dataset of 303 loci successfully extracted from Illumina MiSeq libraries prepared for 143 Onagraceae taxa, plus four outgroups. Specimen collection details including voucher information and determination can be found in Table S1 (Additional file 1).

Although the probe sequences were designed from two species of *Oenothera* sect. *Calylophus*, we did not observe any relationship between target recovery and phylogenetic distance to that section within *Oenothera* (Fig. S1a, Additional file 2) or between target recovery and sample age (Fig. S1b, Additional file 2). To confirm whether genes had paralogous copies in any taxa, we assessed the presence of multiple gene contigs assembled for a gene within each sample using the paralog finding scripts distributed with HybPiper. The distribution of putative paralogs suggests that these duplicate copies are largely recent in origin, potentially impacting some species level relationships (particularly within *Clarkia*) but not the higher-level relationships that are the focus of this study. Our dataset was further supplemented by orthologous 1KP Project transcriptome sequences from 21 species, primarily from *Oenothera* sect. *Oenothera*. The final dataset included 168 accessions (Table S1, Additional file 1). We detected an average of 272 genes across all samples. The number of genes recovered varied from 109 to 309 (mean 272, median 298), resulting in gene sequence matrices ranging from 24 to 99% taxon occupancy (mean 83%, median 85%).

**Fig. 3:**
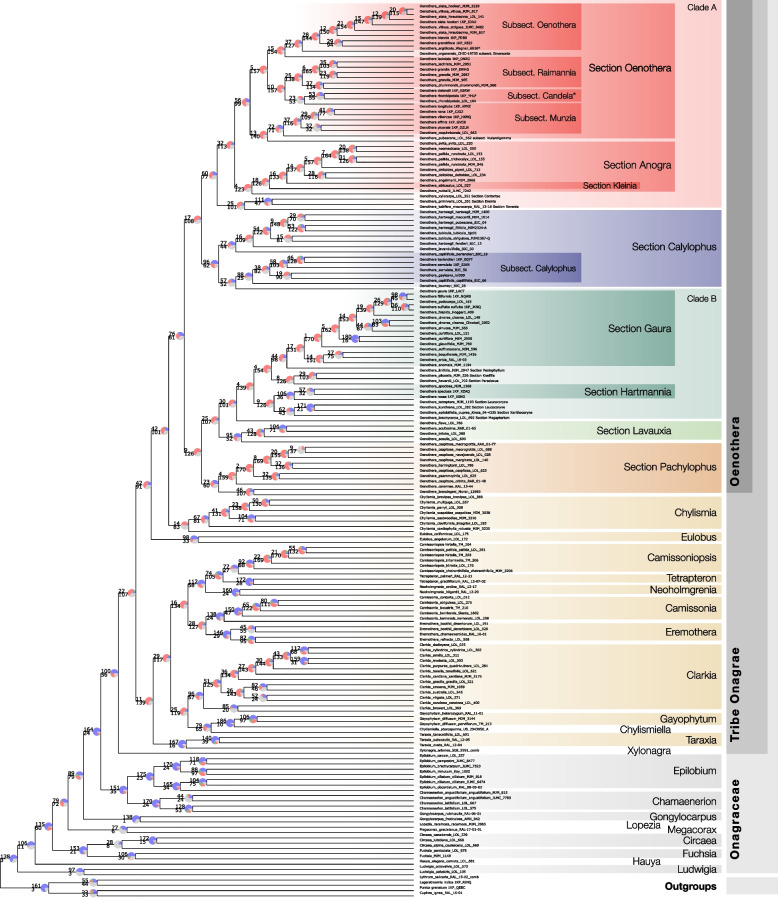
Best-scoring ASTRAL-II tree (displayed as a cladogram) based on 303 input best-scoring maximum likelihood gene trees from RAxML. Numbers above branches represent the number of gene trees in concordance with a particular clade in the species tree (blue in pie chart), whereas those below indicate gene trees in conflict (red/green in pie chart). Pie charts were constructed using 206 genes, and additionally represent the portion of conflicting gene trees that support the main alternative (green), those that support all remaining alternatives (red), and finally those with no information (gray), which could be due to bootstrap support < 50% for the branch in question or missing data (Fig. S1, Additional file 2). The ASTRAL tree and concatenated ML tree are represented with branch lengths (with the exception of tip branch lengths) in Figure S2 (Additional file 2) and individual tree files are provided in the Dryad repository that accompanies this study

We used the individual gene alignments to make two estimations of the species phylogeny by doing the following: 1) concatenating the gene alignments and inferring a species tree using RAxML [25, 26]; and 2) constructing individual gene phylogenies using RAxML and reconstructing the species tree using ASTRAL [27] (Fig. 3), a summary gene tree/species tree method consistent with the multispecies coalescent model.

### Examining gene tree conflict

Large, genome-scale datasets, such as the ones obtained via target capture, have been shown to have very high support via traditional metrics including bootstrapping and posterior probability [28]. Nodes that are maximally supported may still have evidence of conflicting signals among gene trees, which can be further explored by summarizing support for each bipartition across many gene trees. We used PhyParts [29] to assess the number of gene trees concordant with and significantly conflicting with the ASTRAL species phylogeny. As PhyParts requires rooted gene trees, this analysis was done on a reduced set of 206 gene trees that had adequate sampling in our outgroups. Throughout the discussion we will refer to the level of gene tree concordance and conflict accordingly: PhyP = 143/15, referring to the total number of gene trees out of 206 that agree with (143) and disagree with (15) the corresponding topology in the species tree. Note that not all gene trees will be concordant or conflicting; some may be uninformative for a specific bipartition.

**Fig. 4:**
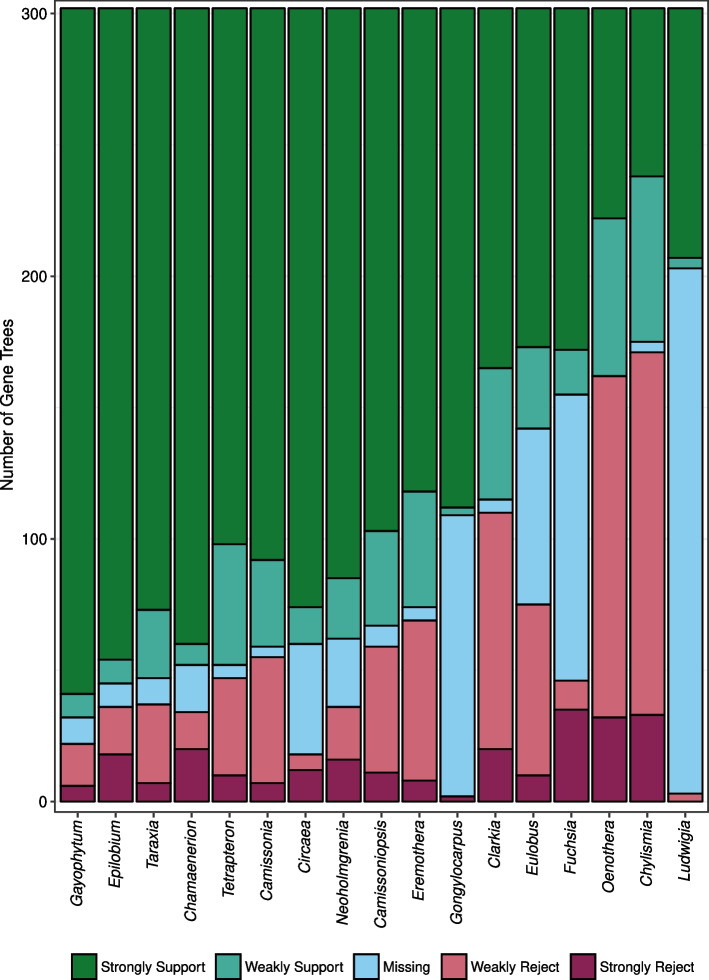
Distribution of support for the monophyly of 17 of 22 genera (x-axis) in Onagraceae among 303 gene trees using DiscoVista. Gene trees (y-axis) are shown to strongly support (dark green), weakly support (teal), weakly reject (pink), or strongly reject (maroon) genera. Shown in blue are gene trees that do not contain any data for a certain genus

**Fig. 5:**
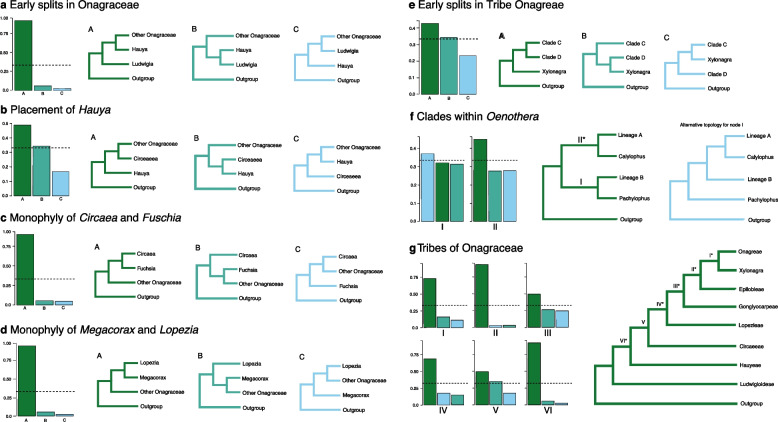
Alternative topologies for clades within Onagraceae. The relative frequency of gene trees is shown for quartets of taxa (bar graphs). Green bars indicate the RAxML-ASTRAL topology, teal and light blue bars indicate alternative topologies. Dotted lines indicate 1/3 of the total gene trees for each quartet; roughly equal proportions for the three possible topologies indicate a polytomy within the present dataset. **a** Early-diverging Onagraceae; **b** Placement of *Hauya*; **c** Monophyly of *Circaea* and *Fuchsia*; **d** Monophyly of *Megacorax* and *Lopezia*; **e** Early-diverging Onagreae, where clades X and Y refer to the following groups: clade X comprises *Camissoniopsis*, *Eremothera*, *Camissonia*, *Tetrapteron*, and *Neoholmgrenia*; clade Y comprises *Clarkia*, *Chylismiella*, and *Gayophytum*; **f** Clades within *Oenothera*, the quartet at node I is ((Lineage B, sect. *Pachylophus*), Lineage A + sect. *Calylophus*) Outgroup) and node II is ((Lineage A, sect. *Calylophus*), (Lineage B + sect. *Pachylophus*) Outgroup), the mode common alternative topology for node I is shown

We further explored the level of support among the gene trees for the monophyly of key clades (Fig. 4) using DiscoVista [30], a software that creates visualizations of discordance in phylogenomic datasets. We also examined the position of historically difficult clades within Onagraceae, comparing the summary (ASTRAL) topology to the two alternative, rooted quartet trees for each focal node (Fig. 5). A dominant summary topology with the two alternative topologies in relatively equal frequency is consistent with speciation in the presence of incomplete lineage sorting (ILS); if all three topologies are present in roughly equal frequencies, this suggests that significant levels of ILS and gene tree estimation error may prevent the accurate resolution of the node for the given data [30].

Our target enrichment approach, therefore, allowed for the construction of a large dataset with minimal missing data (10.24% gaps combined across all trimmed alignments) for loci that contain sufficient variability to be informative from the family to species level. In many cases, areas of the Onagraceae phylogeny that disagreed among previous studies were resolved with higher confidence. In other cases, our results reveal that conflicting prior studies may reflect high levels of gene tree discordance in several key nodes, and that conflict is not restricted to shallow phylogenetic scales [31, 32]. Coalescent theory predicts that in many cases, a gene tree that is concordant with the true speciation history may be less likely than conflicting gene trees [33, 34], and demonstrates that these branches may occur anywhere on the tree, not just in more recent nodes (see Fig. 5f for a clear example of this). This phenomenon is referred to as an anomaly zone, where a set of short branches in a phylogenetic tree can result in the most common gene tree topology differing from the true species tree topology [35].

### Major splits within Onagraceae

The topologies from both the RAxML + ASTRAL analysis and the best-scoring maximum likelihood tree from the concatenated supermatrix are very similar, with relationships between all tribes and genera identical between the two analyses (Figure S2, Additional file 2). All tribal and generic relationships outside Onagreae received 100% support from the ASTRAL and concatenated analyses (Figs. 2, 3 and 4). However, lower-level relationships, especially within the more heavily sampled Onagreae, are often defined by branches with shorter lengths in our ML analysis, as well as increased gene conflict revealed by PhyParts (Fig. 3).

All analyses strongly support the relationship of the pantropical *Ludwigia* (subfamily Ludwigioideae) as sister to the rest of the family (PhyP = 143/15; Fig. 5a), which has been established in previous phylogenetic studies [20, 21, 36–38]. *Ludwigia* also has well-documented morphological autapomorphies, which are floral tube absence, pollen in most sections shed as tetrads (occasionally polyads), a nectary at the base of a stamen, and ovule archesporium single-celled, and outer integument dermal [39, 40]. Previous morphological [41] and molecular phylogenetic ( [21, 38], p. 200) evidence support the monophyly of *Hauya,* but its relationship to other tribes has been difficult to resolve. This moth- or potentially bat-pollinated group of two species (Fig. 1B) ranging from central Mexico to Costa Rica has been hypothesized to possess morphological synapomorphies that closely align *Hauya* with members of *Clarkia*, as well as *Oenothera* sections *Calylophus* and *Gaura * [41, 42]. These hypotheses have been rejected by all molecular phylogenetic analyses [7, 17, 20, 21, 36–38, 43]. Both Ford and Gottlieb [38] and Johnson et al. [17] found support for a branch defining a sister relationship between *Hauya* and tribe Circaeeae. However, we corroborate the result of Levin et al. [21] that *Hauya* is sister to all remaining members of subfamily Onagroideae (A = 100; ML = 100; PhyP = 106/11; Fig. 5b).

The monophyly of *Fuchsia* (A = 100; ML = 100; PhyP = 106/30) and its sister relationship to *Circaea* (PhyP = 153/21; Fig. 5c) is strongly supported, despite striking morphological differences between the two genera. *Fuchsia* (Fig. 1C) is a mainly tropical genus with 4-merous bird-pollinated flowers that are generally red in color, whereas *Circaea* (Fig. 1D) is restricted to northern latitudes and has 2-merous, autogamous, or insect-pollinated white flowers [1]. This sister relationship between the genera is overwhelmingly supported by the analysis of alternative quartets, and the node supporting this relationship shows very little influence of ILS (Fig. 5c). The monophyly of *Circaea* receives high support from both ASTRAL and ML trees (A = 100, ML = 100, Fig. 4). The low number of genes with phylogenetic signal at the node defining the monophyly of *Circaea* is due to the reduced number of genes recovered for sample Circaea_canadensis_LOL_668 (47 of 302 genes). Regardless, the majority of informative genes for this topology agree with the monophyly of *Circaea* (PhyP = 28/8), results that agree with a larger, more comprehensive study of the genus [44].

Johnson et al. [17] found weak support for the placement of *Lopezia* as sister to all members of Onagraceae except for *Ludwigia*. Somewhat similar is a clade of *Lopezia* + *Circaea* + *Fuchsia* obtained in the morphological family-wide phylogenetic study of Hoch et al. [41], but that relationship was based on the single character of integument histogenesis. Neither of these relationships has been recovered in any of the other studies except for Martin & Dowd [45]. Our analyses, however, strongly support the results of Levin et al. [20, 21] and Ford and Gottlieb [38] that tribe Lopezieae (including *Megacorax*) is sister to Gongylocarpeae + Onagreae + Epilobieae (A = 100; ML = 100; PhyP = 79/72; Fig. 5d). Further, we find strong support for a monophyletic *Epilobium* (A = 100; ML = 100; PhyP = 175/23; Fig. 4) and *Chamaenerion* (A = 100; ML = 100; PhyP = 170/24; Fig. 4) and their sister relationship composing Epilobieae (A = 100; ML = 100; PhyP = 151/35), which is consistent with a more detailed study by Baum et al. [46] and others [17, 20, 21]. Tribe Epilobieae is clearly sister to Onagreae, as has been previously reported [17, 20, 21].

### Relationships within tribe Onagreae

The enigmatic Baja California endemic, and presumably hummingbird-pollinated, *Xylonagra arborea* (Fig. 1K) is strongly supported as sister to all other members of Onagreae in some of our analyses (A = 100; ML = 100); however, analysis with PhyParts reveals that only 22 trees support this topology (with 107 informative trees dissenting; Fig. 3). In addition, the DiscoVista relative frequency analysis supports *Xylonagra* as sister to the rest of Onagreae, but there is also moderate support for a sister relationship to the clade comprising *Camissoniopsis*, *Eremothera*, *Camissonia*, *Tetrapteron*, *Neoholmgrenia*, *Clarkia*, *Chylismiella*, and *Gayophytum* (Fig. 5e). The former relationship is consistent with Levin et al. [21] and potentially clarifies previous conflicting results about its placement within the tribe [17, 20]. Inside Onagreae, both the ASTRAL and RAxML analyses strongly support a sister relationship between a clade comprising *Camissoniopsis*, *Eremothera*, *Camissonia*, *Tetrapteron*, and *Neoholmgrenia* (Fig. 2d–f) and a clade comprising *Clarkia*, *Chylismiella*, and *Gayophytum* (Fig. 2a–c) with *Taraxia* as sister to these two clades together. A clade composed of *Clarkia*, *Chylismiella*, *Gayophytum,* and *Taraxia* was recovered with weak supported by Levin et al. [21]; however, Johnson et al. [17] recovered *Taraxia* as sister to all other members of Onagreae, and a relationship of *Clarkia*, *Chylismiella*, and *Gayophytum* as sister to all other remaining members of Onagreae except *Taraxia*. In Levin et al. [21], the relationships of the clade of *Camissoniopsis*, *Eremothera*, *Camissonia*, *Tetrapteron*, and *Neoholmgrenia*, and the clade of *Clarkia*, *Chylismiella*, and *Gayophytum* within the tribe lacked resolution, and weak support was found for inclusion of *Taraxia* within *Clarkia*, *Chylismiella*, and *Gayophytum* as sister to *Clarkia* + *Gayophytum* + *Chylismiella*. The gene tree conflict surrounding the placement of *Taraxia* in the family reveals the source of previous confusion. Although both our ASTRAL and ML analyses give 100% support to the grouping *Taraxia* + (the clade of *Camissoniopsis*, *Eremothera*, *Camissonia*, *Tetrapteron*, and *Neoholmgrenia*, + the clade of *Clarkia*, *Chylismiella*, and *Gayophytum*), only 11 of 139 gene trees are in concordance with this relationship, whereas 11 gene trees also support a relationship of *Taraxia* + (*Eulobus* + (*Chylismia* + (*O.* sect *Pachylophus* + *O*. sect. Lauvaxia + lineage B) + (*O.* sect. *Calylophus* + lineage A))) and 14 gene trees support a relationship of *Taraxia* + *Xylonagra*. This may be a case of an anomaly zone in our current dataset, where the true species tree is not represented by the majority of gene trees [33, 34].

The monophyly of *Neoholmgrenia*, *Camissoniopsis*, and *Tetrapteron* (Fig. 2f–h) is highly supported (A = 100, ML = 100, PhyP = 112/58). However, the node defining the sister relationship *Camissoniopsis* + *Tetrapteron* is highly supported in ML and ASTRAL analyses but received only moderate PhyParts support PhyP = (74/105), with the most common conflicting topology being *Tetrapteron* and *Neoholmgrenia* sister to *Camissoniopsis*. This sister relationship between *Camissoniopsis* and *Tetrapteron* was previously recovered albeit with weak support in Levin et al. [21] and with strong support in Johnson et al. [17]. The remaining two genera of lineage E, *Camissonia* and *Eremothera*, comprise a clade, a result previously suggested or strongly supported in previous analyses. This relationship is poorly supported in our analysis by both ASTRAL (A = 48) and PhyParts (28/127), but the short branch defining this relationship received 100% bootstrap support in our ML analysis. The clade of *Clarkia* + (*Chylismiella* + *Gayophytum*), which was also recovered in Levin et al. [21], receives 100% support in both ASTRAL and ML analyses but is poorly represented by gene trees overall (PhyP = 25/119). However, the sister relationship between *Gayophytum* and *Chylismiella* is supported by higher gene-tree concordance (79/65).

### Relationships within *Oenothera*

As in previous studies [21, 47], we find strong support (A = 100, ML = 100, PhyP = 42/101) across all analyses for the relationship of *Eulobus* as sister to *Oenothera* + *Chylismia* (Fig. 2i–l). Within *Oenothera,* there is strong support among all analyses for the previously described lineages A and B [21]. These deep lineages within the genus were first detected through synapomorphic seed morphology, with lineage A possessing radially enlarged endotestal cells, and lineage B either angled or winged capsules [48]. The monophyly of these lineages was subsequently phylogenetically confirmed, but the placement of the remaining sections (*Calylophus*, *Lavauxia*, *Pachylophus*) has been a mystery, with many conflicting topologies supported with regard to their relationships [17, 20, 21].

*Oenothera* sect. *Calylophus* is a group of 7 spp. and 13 taxa, with a suspected Pleistocene radiation centering around the southwestern U.S., and repeated evolution of both bee pollination (ancestrally hawkmoth-pollinated) and gypsum endemism [23, 49]. This section has previously garnered conflicting phylogenetic support for a sister relationship to lineage B, lineage A, or even to sect. *Pachylophus* [17, 21]. With complete taxon sampling of sect. *Calylophus*, both the ASTRAL and concatenation analyses strongly support a sister relationship of sect. *Calylophus* with lineage A, as does the DiscoVista gene tree analysis (Fig. 5f). Although no representative of sect. *Calylophus* was analyzed in the seed/capsule analysis of Tobe et al. [48], sect. *Calylophus* has since been predicted to be consistent with membership in lineage A, due to its cylindrical (non-angled) capsules [1]. The short branch defining this relationship in our ML analysis and the limited number of gene trees (17) in concordance vs. the 108 gene trees in conflict with this topology warrant caution with this result. Only 17 gene trees agree with our ASTRAL topology; however, there are no alternate topologies supported by more than three gene trees. There is strong support for the monophyly of the two subsections of sect. *Calylophus* (A = 100, ML = 100, PhyP = 96/62) with the exception that *O. toumeyi* ( [23, 49]), traditionally placed in subsect. *Salpingia*, is strongly supported as sister to all other members of subsect. *Calylophus* (A = 100, ML = 100, PhyP = 57/52), corroborating the findings of Cooper et al. [23].

*Oenothera* sect. *Lavauxia*, which has also been historically difficult to place within *Oenothera*, is a widespread hawkmoth-pollinated group ranging from southern Canada to Mexico, with two South American species. The group exhibits striking floral variation: *O. flava*, which is restricted to sky islands in the southwestern U.S. and northern Mexico, exhibits possibly the longest floral tubes in North America [50, 51]; and references therein) despite the modest-sized flowers of geographically widespread conspecifics. We present almost complete taxon sampling of this Sect. (4 of 5 taxa) and find strong support in all analyses for sect. *Lavauxia* as sister to lineage B, corroborating a weakly supported result from Johnson et al. (2009). This relationship was predicted based on the distinctly winged capsules of species in sect. *Lavauxia* [1]. Twenty-five gene trees agree with this topology, and only five gene trees place this section in the lineage containing *Calylophus* + lineage A instead. No other arrangements occurred in more than 3 gene trees.

*Oenothera s*ect. *Pachylophus* is a group of five species and nine taxa with conspicuous, hawkmoth-pollinated flowers that ranges from Canada through the western U.S. to Mexico. Its seeds possess a synapomorphic “collar”, a large, hollow chamber that dramatically imbibes water and has been attributed to its colonization of an impressive habitat range including deserts, dune systems, grasslands, pinyon-juniper woodlands, and coniferous forests [1]. Cladistic analysis of seed coat anatomy suggested an affinity with members of lineage A [48] and previous molecular phylogenetic analyses have left the placement of sect. *Pachylophus* within *Oenothera* either unresolved [21] or weakly supported as sister to lineage A [17]. With complete taxon sampling for sect. *Pachylophus*, we find moderate support in our ASTRAL analysis (A = 85) for a sister relationship between sect. *Pachylophus* and lineage B. However, our ML analysis could not resolve the placement of sect. *Pachylophus*. A deeper exploration of this node reveals nine gene trees in concordance with the ASTRAL topology (i.e., a sister relationship with lineage B), whereas 5 gene trees support a sister relationship of sect. *Pachylophus* to lineage A + sect. *Calylophus*. In addition, the DiscoVista analysis showed relatively equal gene tree frequencies supporting the ASTRAL topology, as well as sect. *Pachylophus* as sister to lineage B, and lineage A + sect. *Calylophus* (Fig. 5f). The patterns of gene tree conflict we observe relative to the summary topology suggest that these relationships fall within the anomaly zone [33, 34], where short times between speciation events and high levels of ILS result in a majority of gene histories that are inconsistent with the species history. The increased taxon and gene sampling of these analyses has revealed underlying conflict in phylogenetic signal among gene trees and confirmed previous difficulties in resolving the phylogenetic affinities of sect. *Pachylophus*. Within sect. *Pachylophus*, the widespread and morphologically diverse species *Oenothera cespitosa* appears paraphyletic as currently defined due to its exclusion of *O. psammophila* and *O. harringtonii*; a result previously suspected based on morphological data [52], and shown recently [22] to be the result of budding speciation arising from edaphic specialization. This more detailed investigation into the taxon relationships in this group has revealed complex relationships among taxa, including potential hybridization [22].

### Relationships within *Oenothera* lineage A

All analyses recover strong support for a sister relationship between *O. xylocarpa* (sect. *Contortae*) and *O. primiveris* (sect. *Eremia*), with high gene concordance (A = 100, ML = 100, PhyP = 111/47). Additionally, ASTRAL analysis supports the inclusion of *O. tubifera* (sect. *Ravenia*) within this clade (A = 97). However, ML analysis recovers a conflicting relationship with *O. tubifera* as sister to all remaining members of lineage A (Fig. 4). Sections *Oenothera* and *Anogra* receive 100% support in both analyses, albeit with only 32 of 113 gene trees in concordance. Subsections within the large section *Oenothera* are generally supported as monophyletic, but with short branch lengths in our ML analysis and high gene conflict reported by PhyParts. Subsections *Oenothera*, *Raimannia*, *Munzia*, and *Candela* all receive 100% support from ASTRAL and ML analyses.

In the only case in our analyses where a topology disagrees with the monophyly of a section, we find evidence that sect. *Anogra* is not monophyletic. In both ASTRAL and ML analyses, sect. *Anogra* forms a strongly supported clade (A = 95, ML = 100) that includes *O. albicaulis* from sect. *Kleinia*, a result previously recovered in phylogenetic analyses focusing on lineage A [53]. The two white-flowered species of sect. *Kleinia* (only *O. albicaulis* is present in our analysis) share several seed morphology characters with subsect. *Raimannia* that are unlike anything in sect. *Anogra* [1, 48]. However, phylogenetic analyses including representatives from both sections have consistently placed *Kleinia* within sect. *Anogra * [53, 54], and have even found evidence that *Kleinia* itself is not monophyletic within sect. *Anogra * [17, 54]. These phylogenetic findings suggest that the aforementioned seed characters, which are both external seed coat features and internal anatomy, are not synapomorphies of the species pair currently circumscribed under sect. *Kleinia* and have potentially been gained or lost more than once within lineage A. The uncertainty with the topology of sects. *Kleinia* and *Anogra* is potentially due to gene tree discordance, or ancient hybrid events that have made the species tree reconstruction challenging. Including *O. coronopifolia*, the other member of sect. *Keinia*, and diploid and tetraploid cytotypes of *O. nuttalii* in future work may help with the reconstruction of this recalcitrant group.

### Relationships within *Oenothera* lineage B

Strong support among analyses was recovered for *O. brachycarpa* (sect. *Megapterium*) as sister to the remainder of lineage B (A = 100, ML = 100, PhyP = 31/101). The monophyly of the large sect. *Gaura* within lineage B is 100% supported by ASTRAL and ML analyses with 44 vs. 98 gene trees in concordance. A sister relationship between sect. *Gaura* and a clade containing sections *Kneiffia*, *Paradoxus*, and *Peniophyllum* received high support in ASTRAL but was defined by a short, weakly supported branch in our ML analysis that is supported by only 4 genes trees (A = 97, ML = 52, PhyP = 4/154). The clade containing sections *Hartmannia* (Fig. 1K), *Leucocoryne,* and *Xanthocoryne* is also strongly supported by ASTRAL and ML analyses (100%), but with only 9 genes of 126 agreeing with this topology.

## Conclusions

Here we present the first phylogenomic analysis of relationships in Onagraceae using 303 nuclear, putatively single-copy genes. Depending on the relationships in question, these increased data resolved relationships that were previously unclear or revealed significant levels of gene tree conflict. Both ASTRAL and RAxML produced virtually identical topologies with regard to the relationships among tribes and genera with high bootstrap support throughout and in many cases high gene tree concordance. However, relationships among genera and sections, especially within the more heavily taxon-sampled Onagreae, reveal high conflict among gene trees for lower-level relationships. These cases, where increased gene number has still failed to confidently resolve relationships within the family reveal deep conflict among gene trees which could be due to rapid radiation, ILS, hybridization (ancient and recent), selection, as well as lack of information content [55–57]. Future analyses must explore these relationships, potentially using increased genomic sampling and lower-level taxonomic case studies.

Genomic sequencing approaches such as HybSeq provide a cost-effective way to gather hundreds of nuclear genes for phylogenetic analysis at multiple phylogenetic scales [58, 59]. With these large multi-gene datasets, however, gene tree conflict presents a significant challenge to species tree inference. Large datasets can help detect patterns of incomplete lineage sorting or hybridization [60, 61], but more data does not always help with estimating a strongly supported bifurcating species tree [62]. In some cases, only a small portion of the genome is unaffected by inter-taxon gene flow or ILS, making it more difficult to determine which loci or genes are the most appropriate for tree inference [31, 35, 62] or whether a bifurcating tree is an accurate representation of the evolutionary history of the group. Gene tree conflict, and the evolutionary mechanisms behind it, are likely causing some of the difficulties in reconstructing relationships within Onagreae [47]. For example, *Xylonagra arborea* is generally supported as sister to the rest of the tribe, but alternative topologies are supported by some analyses [17, 20]. The same is true for *Oenothera* sect. *Pachylophus*. This section has been difficult to place [17, 20, 21], and our DiscoVista analysis indicates the relationships with lineages A and B plus sect. *Calylophus* may fall into the anomaly zone for our current dataset. An important consideration is that speciation may have happened rapidly in these groups, leaving little to no trace of the true evolutionary history [33, 34], or reflecting the fact that a bifurcating tree may not be an accurate representation of a rapid radiation in the presence of gene flow and ILS. We emphasize that the bifurcating species tree presented here represents a hypothesis of relationships rather than the true history, and that the measures of gene tree conflict alongside the tree suggest that evolution was not consistently tree-like throughout the history of the family. Analyzing varying subsets of gene trees under different evolutionary scenarios [62] and using population-level sampling and analysis may be necessary to better elucidate the true evolutionary hisotry of lower-level clades within Onagreae. Understanding the evolutionary history of such groups, whether it be a polytomy or bifurcating tree, is important to provide a contextual building block for further research in ecology and evolution.

## Methods

### Taxon and tissue sampling

Individuals from across Onagraceae were chosen to represent as many lineages as possible, with sampling focused most extensively on tribe Onagreae and *Oenothera*. Leaf material was sampled for 148 individuals from either field-collected (wild specimens), silica-dried tissue, or herbarium vouchers (with a maximum age of 49 years from collection date). Specimen collection details including voucher information, determination, and NCBI SRA accession numbers can be found in Table S1 (Additional file 1). DNA extractions were performed using a modified CTAB protocol [23] involving purification with silica, except in a few cases where repeated attempts resulted in insufficient DNA after the silica cleaning stage, in which case this stage was omitted.

### Library construction, bait capture, sequencing

Genomic libraries with an insert size of 550 bp were prepared using the TruSeq Nano HT DNA Library Preparation Kit (Illumina San Diego, CA, USA) following manufacturer’s instructions, except that all reagent volumes (except PCR reagents) were cut in half beginning with the second addition of AMPure (SPRI) beads (Beckman Coulter, Beverly, MA). Successful library preparation was confirmed with the Qubit 2.0 fluorometer (Invitrogen Carlsbad, CA, USA) using the dsDNA HS Assay Kit, as well as BioAnalyzer 2100 traces (Agilent Technologies, Santa Clara, CA, USA) on a subset of samples. Target enrichment with liquid hybridization was performed using a MYbaits custom target enrichment kit (Mycroarray, Ann Arbor, MI, USA) designed for use in *Oenothera * [22, 23]. Libraries were multiplexed into pools containing 6–18 samples, roughly organized by taxonomic affiliation (e.g., *Oenothera* samples were hybridized together), with 100 ng of total starting library per sample in each pool. In the few cases where less than 100 ng was present, we used the total amount available (lowest successfully attempted ~25 ng). In all cases, we did not exceed 1.2 µg of total DNA per pool as recommended by the manufacturer. Hybridization was performed at 65 °C for ~18 h and enriched library pools were amplified with 14–18 PCR cycles as needed. No correlation was observed between PCR cycle number and ultimate target recovery by sample, suggesting that 18 cycles (or possibly more) results in little target loss through library bias under our multiplexing and sequencing parameters. In many cases, using higher PCR-cycle numbers was crucial for gaining sufficient product concentration for sequencing, especially for samples from older collections and herbarium vouchers. Each resulting PCR-amplified pool was then cleaned with a QiaQuick PCR Purification Kit (Qiagen, Hilden, Germany). Excess adapter, as revealed through the BioAnalyzer, was removed pre-sequencing with a 0.7 to 1 volume ratio of Ampure beads to product. Sequencing of enriched pools of libraries containing 60–80 individual samples was carried out on the Illumina MiSeq System (600 cycle, v3 chemistry) with a final loading concentration of 16.5 pM (estimated from Qubit and BioAnalyzer output) and a 1% molar ratio of PhiX Control (Illumina). Individual sequencing runs resulted in approximately 28 million read-pairs passing Illumina quality filtering with an average of approximately 1–1.5% of reads assigned to each individual. Raw reads are deposited at the NCBI Sequence Read Archive (BioProject ID PRJNA544074); gene alignments, gene trees and species trees, and other related files and codes are deposited at Dryad (https://datadryad.org/stash/share/Um2cZ0ubGDzGAhdXJuXPxzTGy8iL8ceewfVO5yRoLSc).

### Quality filtering, assembly and alignment

Raw, demultiplexed reads from the MiSeq platform were downloaded and quality filtered as paired reads with Trimmomatic [63] using the following settings: ILLUMINACLIP: illumina_adapters.fasta:2:30:10 LEADING:10 TRAILING:10 SLIDINGWINDOW:4:20 MINLEN:20 2. All reads retaining a mated pair were saved for downstream analysis. These data were combined with an additional 21 transcriptomes from the 1KP project (www.onekp.com) for which orthologous genes were assembled from reads using HybPiper, for a final dataset comprising 169 accessions representing 152 taxa and 129 species (including four outgroups in the Lythraceae; Table S1, Additional file 1). To extract exon sequences from raw reads, we used the HybPiper v1.2 pipeline [59]. Briefly, HybPiper searches reads against a file of target gene sequences, assembles reads into contigs with SPAdes [64], aligns contigs to reference targets, and then scaffolds and translates them. For a given target ortholog, both *O. capillifolia* subsp. *capillifolia* and *O. serrulata* sequences were available from the 1KP project, but for many genes only partial exon coverage was available for either of the two species. To avoid issues from samples mapping to only one or the other partial reference sequence, where necessary we created a chimeric sequence representing both species in our HybPiper target file. HybPiper was run with default settings except for specifying –bwa, which uses nucleotide-level data when raw reads are matched to target genes. Due to concerns about sequence divergence affecting gene recovery, for 31 samples outside the tribe Onagreae where less than 275 genes were recovered, HybPiper was rerun using the default BLASTX method that matches reads to targets and aligns SPAdes contigs with targets at the protein level. In five cases, gene recovery improved and for these samples the data based on protein alignment was used instead for downstream analyses. Two python scripts, short_seqs.py and remove_seqs.py (github.com/mossmatters/phyloscripts) were then used to remove gene files in the HybPiper output that represented sequences with < 25% of target sequence length. After these short sequences were removed, samples retained between 47–308 genes (Fig. S1, Additional file 2). For CDS alignments, sequences for a given gene were gathered from all samples into a single FASTA file, with independent files for nucleic and amino acid. Protein coding sequences were searched for stop codons, which were replaced with the letter “X” and these sequences were aligned with MAFFT v7.130b [65] using the following settings: –localpair –maxiterate 1000. Nucleic acid sequences were then mapped to amino acid alignments using pal2nal v14 [66] with default settings. Empty gene files were subsequently removed and positions in alignments which were represented by less than 50% of samples were removed with the alignment trimmer trimAl v1.4.rev.15 (Capella-Gutiérrez et al., 2009).

### Gene tree estimation

Unrooted gene trees with 100 bootstraps each were produced with RAxML-HPC v8.2.0 (Stamatakis, 2014) using partitioning based on codon position, the rapid bootstrap method, the GTRCAT model of nucleotide substitution, and all other parameters on default. In some cases, poor alignments of individual sequences resulted from poor sequence recovery and/or misidentified orthology between HybSeq and transcriptome sequences. To identify poorly aligned sequences, gene trees were searched for branches of unreasonable lengths, defined as branches with lengths exceeding a percentage of the total gene tree depth: 25% for terminal branches, 50% for internal branches, and 75% for outgroup branches. A total of 88 gene trees were flagged by the script brlen_outliers.py (github.com/mossmatters/phyloscripts). After manually investigating each tree, the offending sequence was removed and the corresponding alignments and gene trees were again generated with MAFFT, pal2nal, and RAxML. This manual pruning resulted in 228 sequences that were removed from 137 gene alignments. The distribution of manually removed sequences was such that only 13 samples had their sequences removed from greater than five gene alignments and the maximum number of genes removed for a single sample was 16 genes. After manual pruning, six genes were removed entirely from all analyses due to poor sample representation (contained < 15 sequences) resulting in a final target gene list of 303 genes. Prior to downstream analyses, gene tree branches with < 33% support were collapsed across all gene trees using DendroPy v4.2.0 [67] and sumtrees.py v4.2.0 [68].

To explore an alternative gene tree-building method, we also constructed gene trees for each of the 303 loci using IQ-TREE 1.6.9 and [69], performed model selection with ModelFinder for each locus [70]. To test branch support we used ultrafast bootstrap approximations [71], as well as single branch tests with the approximate likelihood ratio test, both with 1000 replicates [72].

### Species tree estimation

Species tree estimation was carried out in two ways: using ASTRAL [73] to conduct a summary gene tree/species tree analysis and using a concatenated supermatrix. We used the ASTRAL-II implementation (for large datasets) of ASTRAL v4.10.2 with 303 gene trees. Support was evaluated using 100 multilocus bootstrap replicates (which samples from the gene tree bootstraps and accounts for gene tree uncertainty) and the local posterior probability (which evaluates quartet support at each node). This was done separately using the RAxML and IQ-Tree gene trees; the resulting trees were essentially identical. For our concatenated analysis, the 303 genes that passed quality filtering were concatenated into a single FASTA file with 169 samples and a resulting matrix length of 260,466 bases containing 175,265 variable sites. A partition file for this combined alignment was generated using a script distributed with HybPiper (https://github.com/mossmatters/HybPiper/blob/master/hybpiper/fasta_merge.py), which partitioned the alignment by both gene and codon position. A maximum likelihood tree with 100 bootstraps was generated with the same settings specified above for individual gene trees using RAxML HPC v8 [26] on XSEDE using Cipres Science Gateway [74].

### Examining gene tree conflict

Gene tree conflict was examined with PhyParts [29] using the script reroot_trees.py to root gene trees that contained outgroup taxa. This left a remaining subset of 206 gene trees where at least one of our four outgroups were present. An ASTRAL tree was generated with these rooted gene trees using the same parameters as previously mentioned for ASTRAL analysis. This resulting ASTRAL tree was rooted to the outgroups and analyzed with PhyParts in combination with the rooted gene trees. The output from PhyParts was then displayed on the ASTRAL topology (Fig. 3) with phypartspiecharts.py. Note that the remainder of the 206 gene trees not in the first two groups are those with low gene tree support values < 33. The scripts for rerooting gene trees and visualizing the pie charts are available at github.com/mossmatters/phyloscripts.

DiscoVista was used to investigate and visualize phylogenomic discordance. Specifically, the gene tree compatibility and branch quartet frequencies tools were used to look at the monophyly of genera and historically difficult clade topologies, respectively. The monophyly of genera in the ASTRAL species tree was compared to the differing topologies of the 303 gene trees (Fig. 4). The positions of clades within Onagraceae that have been historically difficult to resolve were examined using quartet trees (Fig. 5). DiscoVista analyzes the relative frequency of gene tree topologies that match the species tree topology for a given subset of clades. The ASTRAL tree was compared to alternative topologies for certain clades of interest; if a tree was present in 1/3 or more of gene trees, it was considered the most likely topology. If different topologies were present in roughly equal frequencies, this indicates a hard polytomy in the phylogeny.

### Supplementary Information

**Additional file 1: Table S1.** Table of all samples in the analysis, collecting locations, and where deposited.

**Additional file 2: Figure S1.** (a) Matrix gene recovery per sample for genes with >50% target recovery. Samples are ordered by section of Onagraceae. (b) Matrix of gene recovery ordered by age of sample. **Figure S2.** Tanglegram of ASTRAL species tree (left) and concatenated ML tree (right).

## Data Availability

All DNA sequence data generated for this project can be accessed at the NCBI Sequence Read Archive (SRA), BioProject PRJNA544074. Individual BioSample accession number can be found in Table S1 (Additional file 1). Sequence alignments, tree files, and analysis of discordance file can be accessed via the Dryad Digital Repository: https://datadryad.org/stash/share/Um2cZ0ubGDzGAhdXJuXPxzTGy8iL8ceewfVO5yRoLSc. A supplementary table is presented in Additional file 1 and supplementary figures in Additional file 2.

## Abbreviations

PTH: Permanent translocation heterozygosity
ML: Maximum likelihood
ILS: Incomplete lineage sorting
