# Supplementary material for: A phylogeny of the evening primrose family (Onagraceae) using a target enrichment approach with 303 nuclear loci
Source: BMC Ecol Evol. 2023 Nov 17;23:66. doi: 10.1186/s12862-023-02151-9 (PMC10655384; doi:10.1186/s12862-023-02151-9)
Supplement: Supplementary file 2 — Additional file 2: Figure S1. (a) Matrix gene recovery per sample for genes with >50% target recovery. Samples are ordered by section of Onagraceae. (b) Matrix of gene recovery ordered by age of sample. Figure S2. Tanglegram of ASTRAL species tree (left) and concatenated ML tree (right). [file 12862_2023_2151_MOESM2_ESM.docx]

(a)


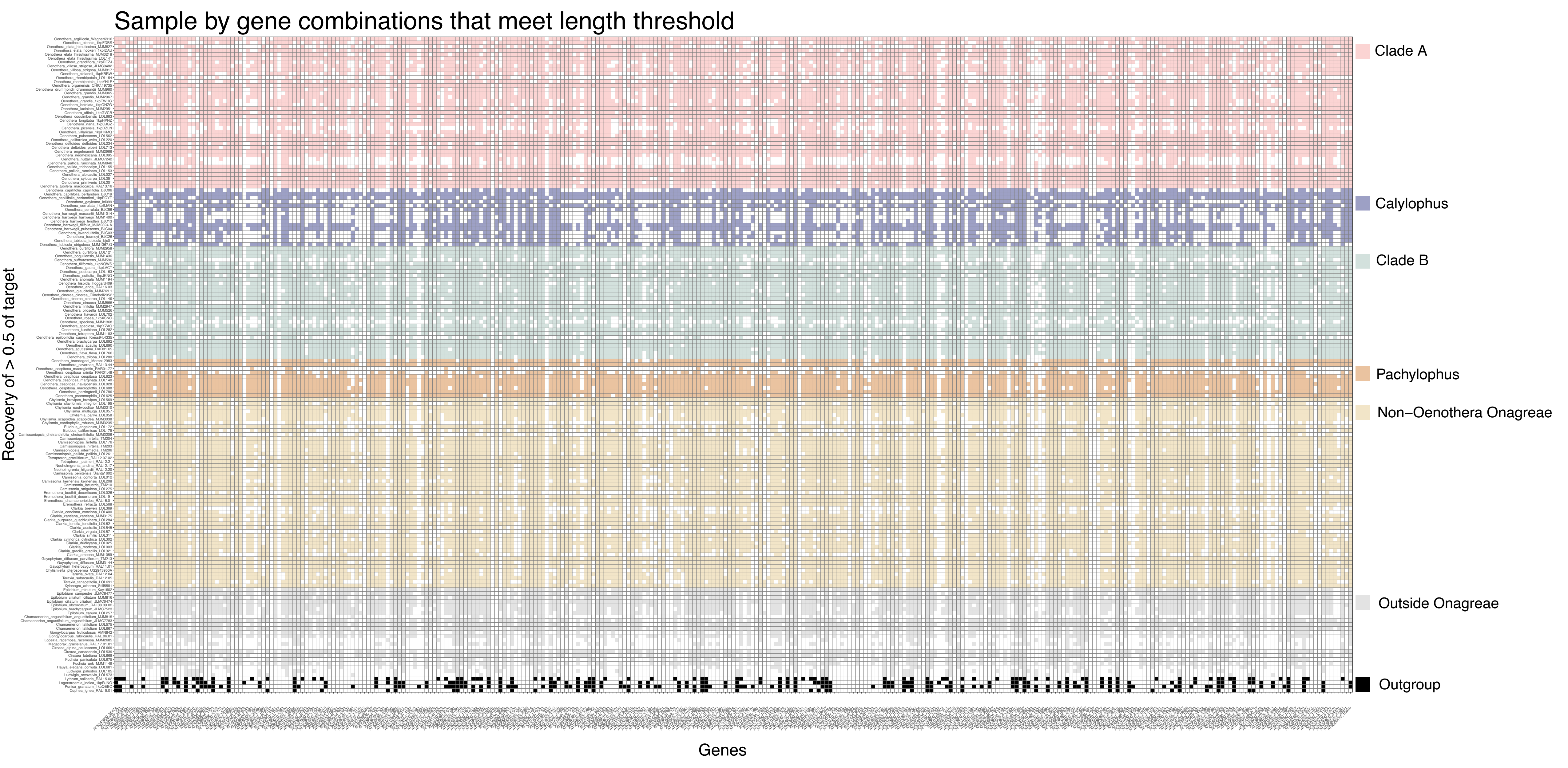

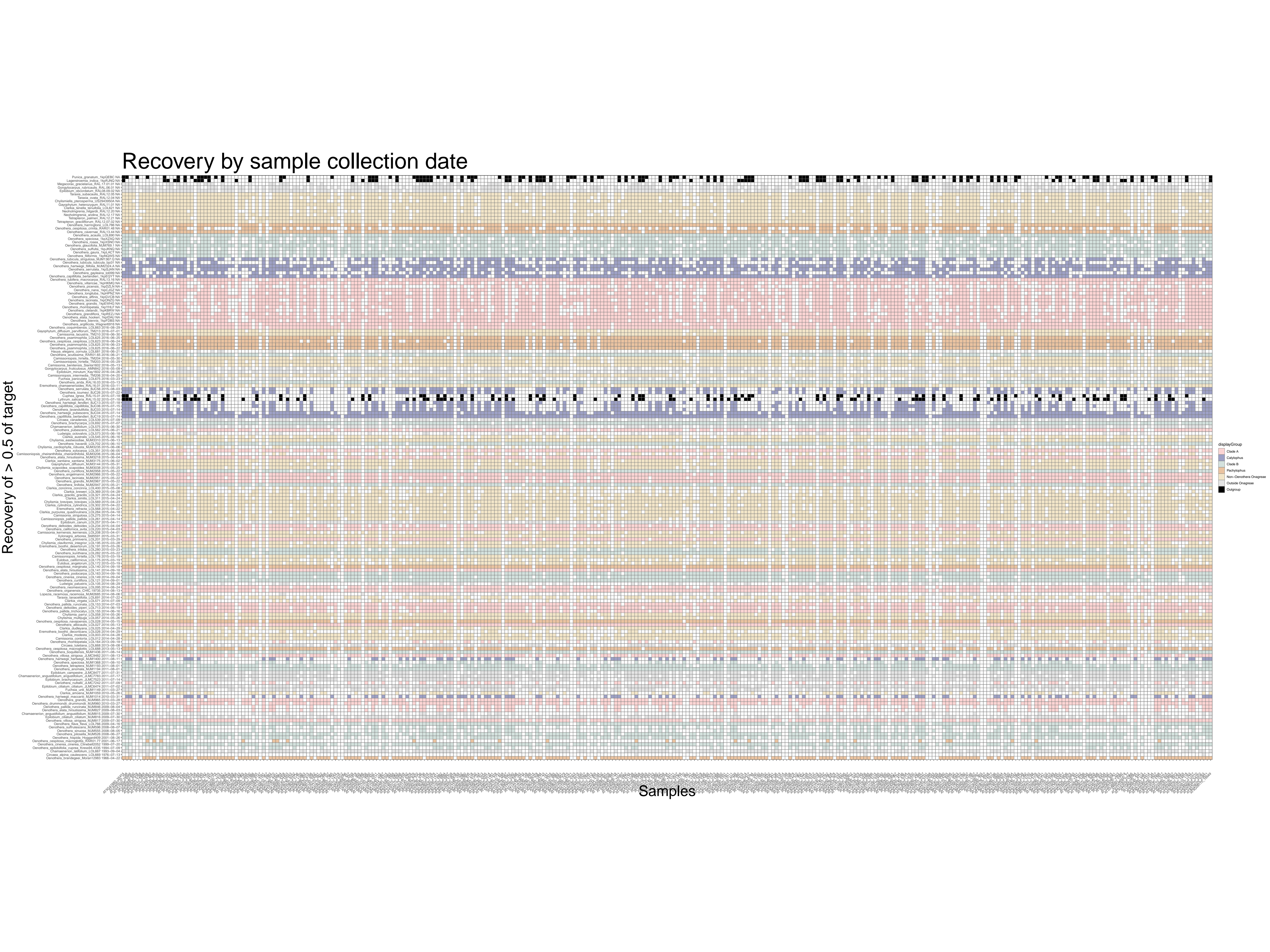
Figure S1. (a) Matrix gene recovery per sample for genes with >50% target recovery. Samples are ordered by section of Onagraceae. (b) Matrix of gene recovery ordered by age of sample

(b)



Figure S2. Tanglegram of ASTRAL species tree (left) and concatenated ML tree (right).
